# Transcriptome RNA Sequencing Data Set of Differential Gene Expression in Escherichia coli BW25113 Wild-Type and *slyA* Mutant Strains

**DOI:** 10.1128/MRA.00294-21

**Published:** 2021-05-13

**Authors:** Olabisi Ojo, Derrick Scott, Bamidele Iwalokun, Babatunde Odetoyin, Anne Grove

**Affiliations:** a Department of Natural Sciences, Albany State University, Albany, Georgia, USA; b Department of Biological Sciences, Delaware State University, Dover, Delaware, USA; c Division of Molecular Biology and Biotechnology, Nigerian Institute of Medical Research, Lagos, Nigeria; d Department of Medical Microbiology and Parasitology, Obafemi Awolowo University, Ile-Ife, Nigeria; e Department of Biological Sciences, Louisiana State University, Baton Rouge, Louisiana, USA; Georgia Institute of Technology

## Abstract

Escherichia coli laboratory strains remain instrumental for the development of tools and techniques in molecular microbiology. The transcriptional regulator SlyA, associated with host-derived oxidative stress, antibiotic resistance, and virulence, is prominent in *Enterobacteriaceae*. Here, we announce a transcriptome data set detailing the global gene expression in E. coli BW25113 and its *slyA* mutant.

## Abstract (precis)

Escherichia coli laboratory strains remain instrumental for the development of tools and techniques in molecular microbiology. The transcriptional regulator SlyA, associated with host-derived oxidative stress, antibiotic resistance, and virulence, is prominent in *Enterobacteriaceae*. Here, we announce a transcriptome data set detailing the global gene expression in E. coli BW25113 and its *sly*A mutant.

## ANNOUNCEMENT

Laboratory strains of Escherichia coli K-12 have been instrumental in the development of molecular tools and techniques in microbiology. Ecological evolutionary developmental biology has revealed remarkable diversity among E. coli strains, especially in the post-genomic era (1). The E. coli K-12-derived strain BW25113 became the parent strain for the Keio collection, a major resource consisting of about 4,000 single-gene deletion mutants (2, 3). However, the complete genome sequence for strain BW25113 remained unavailable until 2014 (4).

The transcriptional regulator SlyA, a member of the multiple antibiotic resistance regulator (MarR) transcription factor family, is often associated with bacterial responses to host-derived oxidative stress, antibiotic resistance, and virulence (5). Previous work has shown that SlyA directly activates the expression of two genes in E. coli K-12 (*hlyE* and *fimB*) by antagonizing H-NS repression, a countersilencing mechanism also reported for other members of *Enterobacteriaceae* (6–8). Microarray-based gene expression profiling of chemostat cultures of an E. coli K-12 MG1655 *slyA* mutant showed no significant differences in transcript abundance compared to wild-type cells, likely because of very low *slyA* expression in wild-type cells, whereas overproduction of SlyA resulted in the identification of additional SlyA targets (6). Here, we present a transcriptome RNA sequencing data set for E. coli BW25113 and its *slyA* mutant.

E. coli BW25113 (wild type) and the *slyA* mutant JW5267-1 were obtained from the Yale University E. coli Genetic Resources Coli Genetic Stock Center (9). The wild-type strain is designated F^−^, Δ(*araD*-*araB*)567, Δ*lacZ*4787(::*rrnB*-3), λ-, *rph-1*; Δ(*rhaD*-*rhaB*)568, *hsdR*514, while the *slyA* mutant is designated F^−^, Δ(*araD*-*araB*)567, Δ*lacZ*4787(::*rrnB*-3), λ^−^, Δ*slyA*720::kan, *rph-1*, Δ(*rhaD*-*rhaB*)568, *hsdR*514. Duplicate biological replicates of each variant were grown to mid-log phase in Luria-Bertani (LB) broth at 37°C with orbital shaking at 200 rpm. Total RNA was prepared using the RNeasy minikit and RNase-free DNase kit (Qiagen). RNA sequencing was performed by Applied Biological Materials (Richmond, BC, Canada). The RNA integrity was verified using an Agilent 2100 Bioanalyzer and the RNA 6000 Pico kit (Agilent). rRNA depletion was carried out using the NEBNext rRNA depletion kit (New England Biolabs). Libraries were generated using the TruSeq stranded mRNA low-throughput (LT) configuration (Illumina). Clustering of libraries was performed using the Illumina cBot system on the NextSeq 2000 (Illumina) sequencer. Sequencing (75-bp paired-end) was performed on the NextSeq 2000 (Illumina) sequencer. FastQC (https://www.bioinformatics.babraham.ac.uk/projects/fastqc) was used for quality control of the data. The Illumina-specific reads and adapters were removed using Trimmomatic (10). The reads were mapped to the E. coli BW25113 reference genome sequence (GenBank accession number CP009273) using STAR (11). Default parameters were used in all analytical software programs. The sequencing yielded 9.9 million to 13.7 million reads per sample with a mapping percentage that ranged from 79% to 85% (Table 1). The analysis supports a previous microarray-based report from a similar E. coli strain, which showed no significant differences in transcript abundance compared to wild-type cells. This data set, with further analysis, incorporating a SlyA-overproducing strain, may facilitate the development of the Keio strain and its *slyA* mutant, which will aid in further understanding SlyA function in E. coli.

**TABLE 1:** Summary of E. coli BW25113 wild-type and *slyA* mutant strain transcriptome sequencing sample data

Sample description^*a*^	Corresponding SRA description	No. of reads	% mapped reads^*b*^	SRA accession no.	GEO accession no.^*c*^
ECWT1.1_rep1	ECWT1_1	2,888,920	79	SRX10348166	GSM5172890
ECWT1.1_rep2	ECWT1_2	2,864,261	79	SRX10348167	GSM5172891
ECWT1.1_rep3	ECWT1_3	2,803,942	79	SRX10348168	GSM5172892
ECWT1.1_rep4	ECWT1_4	2,838,406	79	SRX10348169	GSM5172893
ECWT1.2_rep1	ECWT1_5	3,097,616	79	SRX10348170	GSM5172894
ECWT1.2_rep2	ECWT1_6	3,065,011	79	SRX10348171	GSM5172895
ECWT1.2_rep3	ECWT1_7	3,000,499	79	SRX10348172	GSM5172896
ECWT1.2_rep4	ECWT1_8	3,026,276	79	SRX10348173	GSM5172897
ECMS2.1_rep1	ECMS2_1	2,543,159	82	SRX10348174	GSM5172898
ECMS2.1_rep2	ECMS2_2	2,507,462	82	SRX10348175	GSM5172899
ECMS2.1_rep3	ECMS2_3	2,419,332	82	SRX10348176	GSM5172900
ECMS2.1_rep4	ECMS2_4	2,438,239	82	SRX10348177	GSM5172901
ECMS2.2_rep1	ECMS2_5	3,501,908	83	SRX10348178	GSM5172902
ECMS2.2_rep2	ECMS2_6	3,464,396	84	SRX10348179	GSM5172903
ECMS2.2_rep3	ECMS2_7	3,398,574	83	SRX10348180	GSM5172904
ECMS2.2_rep4	ECMS2_8	3,417,567	85	SRX10348181	GSM5172905

a E. coli BW25113 wild-type samples are denoted ECWT1, and its *slyA* mutant samples are denoted ECMS2. The total number of reads for each of the samples are as follows: ECWT1.1, 11,395,529; ECWT1.2, 12,189,402; ECMS2.1, 9,908,192; and ECMS2.2, 13,782,445.

b Percentage of reads mapped to the reference genome (E. coli BW25113; GenBank accession number CP009273.1).

c The GEO accession number of the transcriptome series is GSE168963, and the SRA BioProject number is PRJNA714667. The GEO title of the project is “Transcriptome RNA Sequencing Data Set of Differential Gene Expression in Escherichia coli BW25113 SlyA Wild Type and Mutant Strains.”

### Data availability.

The transcriptomics data have been deposited in NCBI, and their SRA and GEO accession numbers are provided in Table 1.
